# Implant Migration and Clinical Outcomes in Pediatric Symptomatic Flexible Flatfoot Treated with Subtalar Arthroereisis: A Cohort Study with Long-Term Follow-Up Results

**DOI:** 10.3390/diagnostics15141761

**Published:** 2025-07-11

**Authors:** Yu-Po Huang, Nian-Jhen Wu, Shou-En Cheng, Shang-Ming Lin, Tsung-Yu Lan

**Affiliations:** 1 Department of Orthopedic Surgery, Far Eastern Memorial Hospital, No. 21, Sec. 2, Nanya S. Rd., New Taipei City 220, Taiwan; huangyupomd@gmail.com (Y.-P.H.);; 2 Department of Materials and Textiles, Asia Eastern University of Science and Technology, No. 58, Sec. 2, Sihchuan Rd., New Taipei City 220, Taiwan

**Keywords:** flexible flatfoot, subtalar arthroereisis, implant migration, pediatric orthopedics, long-term outcomes

## Abstract

**Background/Objectives**: Subtalar arthroereisis (STA) is a widely used surgical procedure for symptomatic pediatric flexible flatfoot. However, implant migration remains a concern due to its potential impact on long-term correction and complications. This study evaluated the migration pattern of STA implants and assessed long-term clinical and radiographic outcomes. **Methods**: This retrospective cohort study included 47 feet from children aged 8–13 years who underwent STA with adjunctive soft tissue procedures between 2014 and 2018, following ≥6 months of failed conservative treatment, with a minimum follow-up of 5 years. Exclusion criteria included neuromuscular or rigid flatfoot. Weight-bearing radiographs assessed anteroposterior (AP) and lateral Meary’s angles, reflecting forefoot-to-hindfoot alignment, and calcaneal pitch, indicative of longitudinal arch height. Implant migration was recorded and clinical outcomes were measured by the American Orthopedic Foot and Ankle Society (AOFAS) score. Measurements were recorded preoperatively, immediately postoperatively, and at 1 month, 3 months, 6 months, 1 year, and 5 years. **Results**: Radiographic correction was significant and sustained at 5 years. The AP Meary’s angle improved from 13.09° to 5.26° at 1 month and 6.69° at 5 years (*p* < 0.001); lateral Meary’s angle from 9.77° to 4.06° and 4.88° (*p* < 0.001); and calcaneal pitch from 14.52° to 16.87° and 16.89° (*p* < 0.001), respectively. AOFAS scores increased from 67.52 to 90.86 at 1 month and 96.33 at 5 years (*p* < 0.001). Implant migration peaked within the first postoperative month (mean: 3.2 mm on ankle AP view; 3.0 mm on foot AP view) and stabilized thereafter. Four cases of complications included implant dislodgement, subsidence, and persistent sinus tarsi tenderness, which were successfully resolved after appropriate management. No recurrence of deformity was observed. **Conclusions**: STA implant migration is most pronounced during the first month, likely due to physiological settling as the foot adapts to altered biomechanics. With appropriate implant selection, technique, and follow-up, migration does not compromise long-term correction or outcomes. In general, symptomatic cases can often be managed conservatively prior to implant removal.

## 1. Introduction

Flatfeet, also known as pes planus or planovalgus, are commonly observed in children aged >8 years [1,2]. Flexible flatfoot is primarily characterized by medial longitudinal arch collapse, forefoot abduction, and hindfoot valgus resulting from excessive eversion of the subtalar joint. Most individuals with flexible flatfeet remain asymptomatic [3,4,5,6].

Most pediatric flexible flatfeet cases self-recover with the development of ligaments, bones, and neuromuscular functions [1,7,8,9]. Some adolescents with flexible flatfoot experience midfoot pain and are affected by decreased lower limb function, resulting in reduced quality of life [10,11]. In addition, medial column instability, Achilles tendon contracture, gastrocnemius aponeurosis tightness, and peroneus muscle spasms are observed in patients with flatfoot.

Although the standard management of flexible flatfoot remains controversial, surgery is indicated for patients with foot deformity, midfoot pain, restricted ankle range of motion, gait changes, and excessive fatigue during activity [12].

Surgical options for flatfoot include tendon procedures, osteotomies, subtalar arthroereisis (STA), and arthrodesis. Pure soft tissue procedures yield suboptimal correction [4]. While lateral column lengthening and medial calcaneal osteotomy effectively address forefoot abduction and hindfoot valgus, they may result in malunion and prolonged recovery, without fully correcting subtalar deformity. Arthrodesis is contraindicated in pediatric cases due to risks of early arthritis and loss of joint cushioning [4,13,14].

Subtalar arthroereisis implantation is a minimally invasive, rapid-recovery, three-dimensional corrective procedure that restricts excessive ankle joint eversion [15,16]. Even though STA is used worldwide, the correction effect remains controversial because of the small sample size and lack of long-term follow-up [17,18]. Radiographic and functional outcomes are complementary, and both are essential for a comprehensive evaluation of surgical efficacy [19,20]. However, Suh et al. pointed out the disadvantages of the STA, including persistent sinus tarsi pain, implant migration, and overcorrection [21]. Black et al. reported persistent pain in 36.4% of patients who received STA treatment [22], and Smith et al. found a 19.3% rate of postoperative implant migration [23]. Notably, most previous studies addressing implant migration have not provided detailed analyses of its temporal progression during long-term follow-up, thereby limiting understanding of implant behavior over time. This study aims to quantify implant migration trends and their associations with postoperative complications, as well as to assess the long-term radiographic and functional outcomes of pediatric flexible flatfoot treated with STA—addressing a significant gap in the current literature.

## 2. Materials and Methods

### 2.1. Criteria and Patient Selection

This retrospective study was approved by the Institutional Review Board (ethics committee approval number: 113195-E, approval date: 27 August 2024). Patients were identified using institutional surgical and imaging databases. To protect patient privacy, all data were deidentified.

We retrospectively reviewed all patients between 8 and 13 years who underwent STA for symptomatic flexible flatfoot at our institution between 2014 and 2018. All procedures were performed by a single senior pediatric foot and ankle orthopedic surgeon (Tsung-Yu Lan, MD, PhD). Patient eligibility was determined based on the following criteria:

Diagnosis and Indication: Radiographic evidence of flexible flatfoot in weight-bearing views, characterized by the collapse of the medial longitudinal arch, forefoot abduction, and hindfoot valgus, accompanied by activity-related pain or fatigue of the foot or lower limbs.

Age: Between 8 and 13 years at the time of surgery.

Failure of Nonoperative Management: Persistent symptoms or progression of deformity despite ≥6 months of structured conservative treatment, including custom foot orthoses, physiotherapy, and targeted exercise.

Follow-up: Availability of clinical and radiographic data for a minimum of 5 years postoperatively.

Patients with neuromuscular or neurological disorders (e.g., cerebral palsy) that could affect the natural history or response to treatment of flatfoot were excluded.

### 2.2. Surgical Technique and Postoperative Management

**Figure 1:**
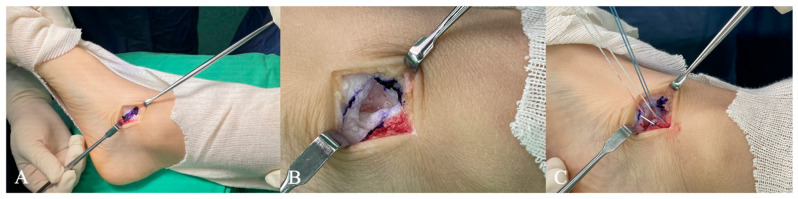
The surgical technique of talonavicular joint capsule plication. (**A**) A linear incision was made on the medial side of the talonavicular joint, and a longitudinal window was marked. (**B**) The joint capsule window was incised and reflected to expose the talonavicular articulation. (**C**) Each capsule margin was reinforced with two non-absorbable braided sutures, which were then tied to imbricate the capsule with the foot in an inverted position, creating a medial arch.

All procedures were performed under general anesthesia with the patient in the supine position. Prophylactic intravenous antibiotics were administered preoperatively, and a pneumatic tourniquet was inflated to 200 mmHg. A positive preoperative Silfverskiöld test indicated the need for concomitant gastrocnemius recession, which was performed using the Strayer technique [24,25]. In patients with a clinically visible accessory navicular, a Kidner procedure was performed [26,27]. All other patients who did not undergo the Kidner procedure received talonavicular joint capsule plication to reinforce medial column stability (Figure 1).

An incision was made over the sinus tarsi, and dissection was carried out through the deep fascia and joint capsule to expose the sinus tarsi tract. A guidewire was inserted into the tract under direct visualization. Trial implants were sequentially introduced along the guidewire and temporarily positioned to assess hindfoot alignment and subtalar joint motion. The appropriate implant size was defined as the one that reduced subtalar joint excursion by approximately 50% compared with the preoperative range and corrected hindfoot valgus to a neutral position. Once optimal positioning and correction were confirmed, an implant (BIOARCH^®^, Wright, TN, USA) was inserted. The implant tip was advanced to a position near the longitudinal talar axis.

Postoperatively, a short leg cast was applied immediately in the operating room and maintained for 2 weeks. Full weight-bearing was allowed once the patient returned to the ward, with walker assistance for 1–2 weeks depending on pain tolerance and individual recovery [28]. Routine follow-up evaluations were conducted in the outpatient clinic at 1, 3, 6, and 12 months and at 5 years postoperatively. At each visit, clinical outcomes and radiographic assessments were performed to monitor implant stability, maintenance of alignment, and symptom resolution.

### 2.3. Clinical and Radiological Evaluation

Prior to surgery, a comprehensive evaluation was performed, including detailed medical history documentation and thorough physical examination. Ankle function was clinically assessed using the American Orthopaedic Foot and Ankle Society (AOFAS) ankle–hindfoot scale, a standardized 100-point scoring system. Radiographic evaluation was conducted under standardized weight-bearing conditions, measuring the anteroposterior (AP) Meary’s angle, lateral Meary’s angle, and calcaneal pitch [29]. These radiographic parameters were recorded preoperatively and reassessed at designated postoperative follow-up intervals.

**Figure 2:**
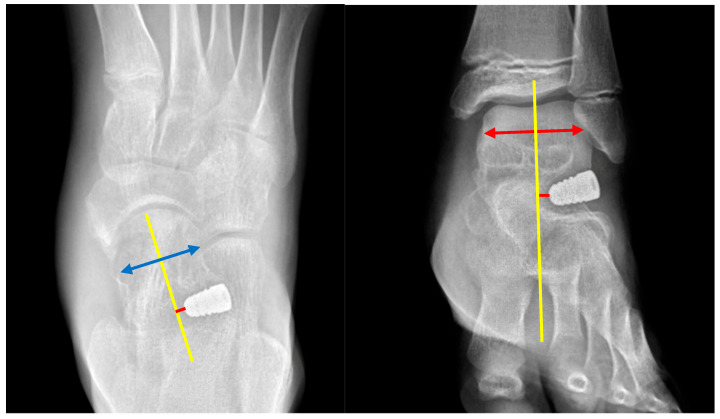
An assessment of implant positioning on anteroposterior radiographs. The yellow line represents the longitudinal axis of the talus. The double-headed blue arrow indicates the width of the talar neck in the foot AP view, and the double-headed red arrow indicates the width of the talar body in the ankle AP view. The red line indicates the perpendicular distance from the implant tip to the talar axis.

The implant position was evaluated using AP foot and ankle radiographs. The talar axis served as the reference line to divide the field into medial and lateral quadrants. On the AP foot view, implant tips extending medial to the talar axis were classified as negative, and those extending lateral were classified as positive. The same criterion was applied to the AP ankle view: implant tips located medial to the talar axis were assigned a negative value, while those lateral to it were assigned a positive value (Figure 2). We also calculated the migration distance in the following radiographs.

### 2.4. Statistical Analysis

Descriptive statistics for the implant migration distance were reported as mean and interquartile range. Repeated-measures comparisons of migration distance across follow-up intervals were performed using the Friedman test. Radiographic parameters (AP and lateral Meary’s angles and calcaneal pitch) were presented as the mean ± standard deviation (SD). Changes in radiographic measurements between the time points were evaluated using the Wilcoxon signed-rank test. All analyses were conducted using SPSS software (version 26.0; IBM Corp., Armonk, NY, USA). Statistical significance was set at *p* < 0.05. A post hoc power analysis was conducted to estimate the statistical sensitivity of the Friedman test. To evaluate intra-observer reliability, a subset of 20 randomly selected radiographs were independently measured twice by one observer.

## 3. Results

### 3.1. Patient Demographics and Clinical Characteristics

We retrospectively analyzed data from 47 feet in patients aged 8–13 years at the time of operation with flexible flatfoot who underwent subtalar arthroereisis (STA) combined with adjunctive soft tissue procedures at the Far Eastern Memorial Hospital between January 2014 and December 2018. All patients underwent regular follow-ups for 5 years, and all clinical and radiographic data were accessible. Patients with neuromuscular diseases, rigid flatfoot, and previous flatfoot surgical treatment or those who were lost to follow-up were excluded. The mean follow-up duration was 282 ± 16 weeks.

**Figure 3:**
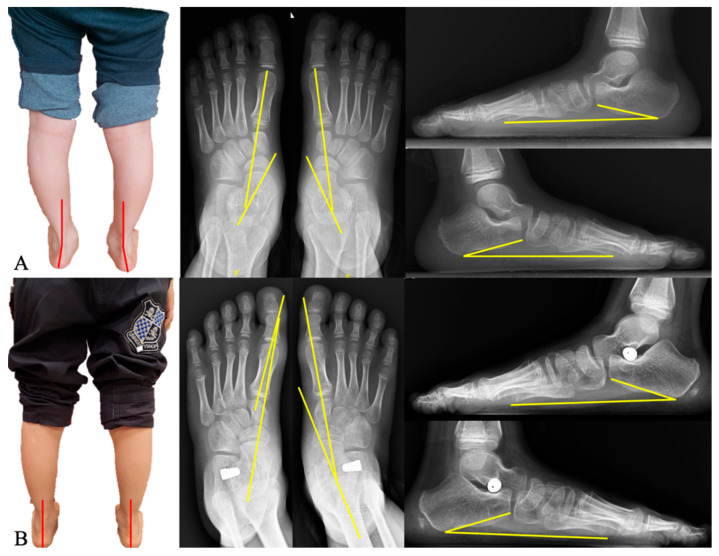
An 8-year-old boy with bilateral flatfoot presenting with hindfoot valgus and forefoot abduction. Red lines on clinical photos illustrate hindfoot alignment in coronal plane. Yellow lines on AP and lateral radiographs represent Meary’s angles, and calcaneal pitch, respectively. (**A**) Preoperative clinical photograph reveals hindfoot valgus, while radiographs demonstrate an increased Meary’s angle and reduced calcaneal pitch, indicative of pes planovalgus deformity. (**B**) Postoperative clinical image shows the restoration of heel alignment, accompanied by radiographic correction of key parameters.

The mean age of the patients was 11.1 ± 1.29 years (range: 8–13 years old). The cohort included 39 male and 8 female feet, demonstrating a significant sex distribution difference (*p* < 0.001). Flatfoot involved bilateral feet in 21 patients (18 males and 3 females), the right foot only in 3 patients, and the left foot only in 2 patients (*p* = 0.52; Table 1, Figure 3).

All patients achieved excellent wound healing without infection. The American Orthopaedic Foot and Ankle Society (AOFAS) score increased from 67.52 ± 6.21 points preoperatively to 90.86 ± 3.07 at 1 month postoperatively, further rising to 93.15 ± 2.21 at 1 year and 96.33 ± 8.15 at the 5-year follow-up (*p* < 0.001).

### 3.2. Radiological Outcomes

For flatfoot correction effect, the AP Meary’s angle decreased from 13.09 ± 7.19° preoperatively to 4.79 ± 4.58° postoperatively (*p* < 0.001). The follow-up AP Meary’s angles at 1 month, 3 months, 6 months, 1 year, and 5 years were 5.26 ± 4.08°, 6.03 ± 4.18°, 6.32 ± 4.27°, 6.83 ± 4.80°, and 6.69 ± 4.46°, respectively, indicating a significant maintenance of the corrective effect. (*p* < 0.001). The AP Meary’s angle increased from 4.79 ± 4.58° to 6.69 ± 4.46° between the postoperative and fifth-year follow-up (*p* = 0.002).

The lateral Meary’s angle decreased from 9.77 ± 5.37° before the operation to 4.13 ± 3.13° after the operation (*p* < 0.001). The follow-up lateral Meary’s angles at 1 month, 3 months, 6 months, 1 year, and 5 years were 4.06 ± 3.12°, 4.34 ± 3.23°, 5.10 ± 3.97°, 4.86 ± 4.25°, and 4.88 ± 4.31°, respectively (*p* < 0.001). The calcaneal pitch angle increased from 14.52 ± 3.19° before the operation to 17.45 ± 2.23° after the operation. The follow-up calcaneal pitch angles at 1 month, 3 months, 6 months, 1 year, and 5 years were 16.87 ± 2.19°, 16.65 ± 2.31°, 16.64 ± 2.56°, 16.68 ± 2.50°, and 16.89 ± 2.50°, respectively (*p* < 0.001). All three radiographic parameters showed statistically significant correction of flatfoot at each postoperative time point (Table 2).

### 3.3. Implant Position and Migration

On the subject of implant position on ankle AP view, 46 of 47 implants (97.9%) were positioned medial to the talar axis, while only 1 implant (2.1%) was located laterally immediately after surgery. At subsequent follow-up, 38 implants (80.9%) exhibited lateral migration, whereas 9 implants (19.1%) demonstrated medial migration, indicating a predominant trend toward lateral displacement over time. The mean implant position on the ankle AP view at each time point was as follows: −4.46 mm immediately postoperative, −1.55 mm at 1 month, −1.77 mm at 3 months, −1.73 mm at 6 months, −1.74 mm at 1 year, and −2.05 mm at 5 years. On foot AP view, 34 implants (72.3%) demonstrated lateral placement relative to the talar axis at the immediate postoperative stage, defined as a positive implant position. In contrast, 13 implants (27.7%) were at negative positions. During follow-up, 40 of 47 implants (85.1%) exhibited lateral migration, while 7 implants (14.9%) presented medial migration, again with a trend toward lateral migration. The mean implant positions on foot AP view were +1.17 mm at 1 month, +2.89 mm at 3 months, +3.21 mm at 6 months, +3.40 mm at 1 year, and +3.67 mm at 5 years.

Regarding implant migration, the mean shift observed on the ankle AP radiograph was 3.24 mm from the immediate postoperative period to 1 month, 0.93 mm from 1 to 3 months, 0.49 mm from 3 to 6 months, 0.51 mm from 6 months to 1 year, and 0.78 mm from 1 to 5 years. On the foot AP view, the corresponding migration distances were 3.01 mm, 1.21 mm, 0.54 mm, 0.64 mm, and 0.76 mm, respectively. The greatest migration occurred within the first month postoperatively, which was statistically significant on both ankle and foot radiographs (Table 3; *p* < 0.001), with a post hoc statistical power of 94.9%.

Intraclass correlation coefficients (ICCs) were calculated to assess interobserver reliability, demonstrating excellent agreement for all radiographic measurements: AP Meary’s angle (ICC = 0.84; 95% CI, 0.78–0.90), lateral Meary’s angle (ICC = 0.86; 95% CI, 0.82–0.90), calcaneal pitch (ICC = 0.87; 95% CI, 0.82–0.92), implant migration distance on ankle AP view (ICC = 0.85; 95% CI, 0.78–0.92), and implant migration distance on foot AP view (ICC = 0.88; 95% CI, 0.83–0.93).

### 3.4. Complications

**Figure 4:**
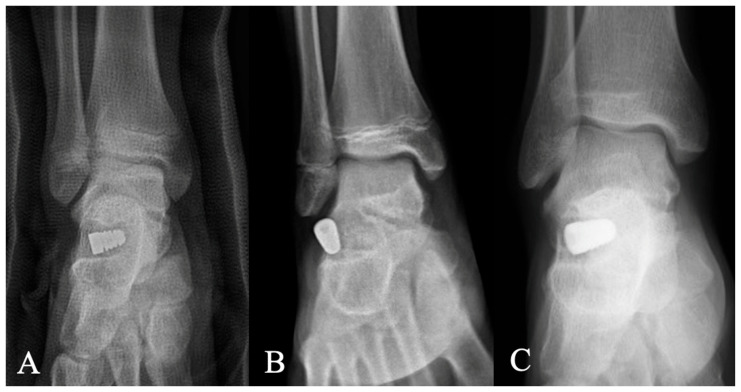
Early implant dislodgement in a 13-year-old boy who sustained an ankle sprain 1 month postoperatively. (**A**) An immediate postoperative AP radiograph demonstrating appropriate implant placement. (**B**) A follow-up radiograph at 1 month showing dislodgement of the implant with loss of correction. (**C**) Five-year radiographic follow-ups showed the implant in a good position.

**Figure 5:**
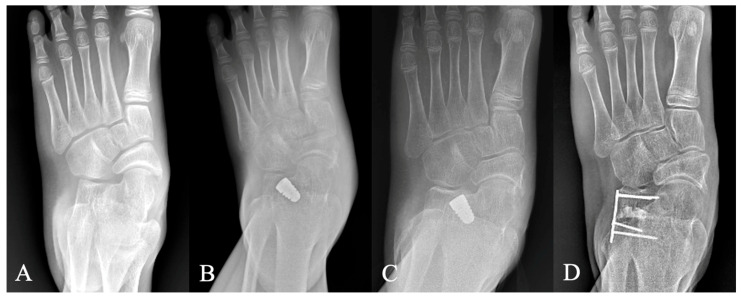
Implant subsidence in a 10-year-old girl who underwent STA and later sustained an ankle sprain. (**A**) A preoperative AP radiograph of the left foot showing forefoot abduction deformity. (**B**) An AP radiograph at 7 months postoperatively reveals subsidence of the STA implant following a sprain injury. (**C**) A radiograph taken 1 year postoperatively also demonstrates implant subsidence. (**D**) Bone union was achieved following revision surgery with lateral column lengthening.

Complications were observed in four feet (Table 4). One patient experienced a low-energy ankle sprain one month postoperatively, resulting in early implant dislodgement. Revision STA with re-implantation was performed successfully, with no subsequent sequelae (Figure 4). The second complication involved implant subsidence in a 10-year-old girl who had undergone bilateral STA combined with the Kidner procedure. Seven months postoperatively, she sustained a sprain injury; radiographs revealed sinking of the left implant and the development of a fixed planovalgus deformity. She subsequently underwent lateral column lengthening, achieving bone union one year after surgery (Figure 5).

Persistent sinus tarsi discomfort occurred in two patients. One underwent implant removal at one year postoperatively, with maintenance of hindfoot and forefoot alignment at the latest follow-up. The other was successfully treated with a corticosteroid injection, resulting in complete resolution of symptoms.

## 4. Discussion

Subtalar arthroereisis (STA) is most commonly indicated for symptomatic flexible flatfoot in children, particularly in those who continue to experience pain or dysfunction despite conservative treatment [30,31]. Unlike in adults, most pediatric cases of flatfoot are asymptomatic, making it challenging to establish clear surgical indications [30,32]. In clinical practice, suitable candidates for STA are children with symptomatic deformities, such as foot pain, fatigue, or activity-related limitations, that do not respond to orthotic use, stretching, or activity modification [31,33]. The primary treatment objective in pediatric patients is to correct forefoot abduction and hindfoot valgus deformities while guiding the developing foot toward a more physiological arch configuration, all without compromising ankle joint mobility. Mazzotti et al. proposed an optimal age window of 9 to 12 years for STA in children, as this period may offer the greatest potential for skeletal remodeling during growth [34].

Both surgeons and patients should consider the corrective potential of STA in the treatment of pediatric flexible flatfoot. Clinical and radiographic outcomes in this population have been consistently favorable [35]. Wang et al. reported sustained correction 9 to 10 years postoperatively, emphasizing that restoration of the medial arch and correction of forefoot abduction are key determinants of functional success [19]. A recent 10-year follow-up study involving 38 feet in 19 pediatric patients demonstrated significant improvements in the talonavicular coverage angle, Meary’s angle, and talar declination angle, with 71% of feet achieving normal arch alignment and 84% of patients reporting good to excellent outcomes on the AOFAS scale [19].

In our cohort of 47 pediatric patients with symptomatic flexible flatfoot treated with STA, we observed early and substantial improvements in both radiographic alignment and clinical function, with no recurrence of deformity at the 5-year follow-up. The AP Meary’s angle, lateral Meary’s angle, and calcaneal pitch all showed statistically significant improvement both immediately postoperatively and at 5 years. These findings support the efficacy of STA in achieving durable correction of pediatric flatfoot. The sustained improvement in AOFAS scores reflects not only pain relief but also enhanced functional capacity, enabling most children to return to full participation in sports and daily physical activities.

Migration is typically defined radiographically as displacement of the implant from its intended position within the sinus tarsi. Previous studies have reported migration rates as high as 19.3%, particularly with undersized or malpositioned implants [23]. Clinically, implant migration may present with persistent sinus tarsi pain, implant prominence, loss of correction, or, in severe cases, implant extrusion [33,36,37,38]. Persistent sinus tarsi pain has been associated with inflammatory reactions, such as reactive synovitis or foreign body granuloma [39].

Metcalfe et al. reported that early return to high-impact activities postoperatively significantly increases the risk of STA implant migration [36]. In more severe cases, chronic mechanical stress from a migrated implant may lead to osseous complications. Corpuz et al. described a case of talar neck fracture occurring a decade after STA, attributed to abnormal force transmission caused by long-standing implant malposition [40]. However, despite these observations, the temporal progression and patterns of implant migration remain poorly characterized in the current literature.

Our study highlights that in pediatric patients, ongoing skeletal growth may contribute to subtle shifts in implant position over time. However, in most cases, surrounding soft tissues appear to remodel and stabilize the implant, which may achieve its optimal position through early migration. The greatest degree of implant migration occurred within the first postoperative month, followed by relative positional stability that persisted through the 5-year follow-up. While this pattern may reflect physiological settling, such interpretation remains speculative and warrants further investigation.

Future studies incorporating biomechanical modeling or advanced imaging modalities such as weight-bearing CT are warranted to better substantiate this claim and further explore the potential for rotational migration. Moreover, skeletal growth during follow-up may alter radiographic landmarks, potentially introducing measurement bias and contributing to interobserver variability. These factors should be addressed in subsequent research. Variations in implant design may also influence migration behavior and should be considered when comparing outcomes across studies.

The management of implant migration depends on the severity of symptoms and the degree of positional change. Asymptomatic migration, particularly if radiographically minor and the foot remains corrected, may not require intervention. However, implant removal or revision is warranted if implant prominence, dislodgement, or functional deterioration occurs. Notably, Cook et al. investigated the risk factors associated with explantation after STA and reported that patients who underwent implant removal had significantly higher odds of radiographic undercorrection [41].

Symptomatic migration with irritation either resolved spontaneously within 1 year or was managed with local corticosteroid injections in our cohort. One patient experienced early implant dislodgement secondary to an ankle sprain, and another developed implant subsidence following a sprain injury at 7 months postoperatively. Neither case was associated with early implant migration. Notably, only one patient in our series exhibited both early implant migration and persistent sinus tarsi pain and underwent implant removal at the 1-year postoperative follow-up.

This study has several inherent limitations due to its retrospective, single-center design, which may introduce potential sources of bias. Selection bias may have occurred due to non-randomized case inclusion, and unmeasured confounders could not be fully controlled. Information bias may also possible given the reliance on retrospective medical record review. Although strict inclusion criteria and standardized surgical techniques were employed, the absence of a control group limits direct comparisons with alternative surgical methods or conservative management. A control group was not included because the study focused on radiographic migration and long-term outcomes of STA in a homogenous surgical cohort. Patients who responded to conservative treatment were not surgical candidates, and alternative procedures were not routinely performed at our institution during the study period. Additionally, multiple comparisons across follow-up time points may increase the risk of Type I error. Given the exploratory nature of the analysis, Bonferroni correction was not applied, and findings should be interpreted with appropriate caution.

Second, implant migration was assessed using standard AP radiographs, which may not capture subtle three-dimensional changes or rotational shifts within the sinus tarsi. Advanced imaging techniques, such as computed tomography (CT) or weight-bearing cone-beam CT, can provide more precise assessments to enhance assessment accuracy.

Third, all surgeries were performed using a single implant system (BIOARCH^®^), and the findings may not be generalizable to other implant types with different designs or biomechanical profiles.

Lastly, while our 5-year follow-up provided meaningful long-term data, outcomes beyond skeletal maturity in children are needed to assess the durability of correction and any potential late complications such as subtalar joint degeneration or delayed recurrence. Additionally, the method used to assess implant migration may be subject to minor variability owing to changes associated with patient growth over time.

## 5. Conclusions

Subtalar arthroereisis combined with adjunctive soft tissue procedures appears to be a promising surgical option for managing symptomatic pediatric flexible flatfoot in this cohort. Although early implant migration was most commonly observed within the first postoperative month, it did not correlate with clinical symptoms or adversely affect short- to mid-term outcomes. However, given the retrospective design, limited sample size, and radiographic measurement constraints, these findings should be interpreted with caution. Further prospective studies incorporating advanced imaging and longer follow-up are warranted to clarify the clinical significance of early migration and inform patient-specific management strategies.

## Figures and Tables

**Table 1:** Baseline characteristics of patients.

	Total (n = 47)	*p* Value
Age (years) Mean ± SD Range	11.1 ± 1.29 8–13	
Gender (Male–Female)	39:8	**<0.01**
Affected side (Left–Right)	23:24	0.52
Bilateral foot cases	21	
Soft tissue procedure		
Medial capsule plication	31 (66%)	
Kidner procedure	16 (34%)	
Gastronomes lengthening (Strayer)	25 (53%)	

Note: Statistically significant *p* values are shown in bold. SD, standard deviation.

**Table 2:** Radiographic parameters for flatfoot correction.

Timepoint	AP Meary’s Angle		Lateral Meary’s Angle		Calcaneal Pitch	
	Mean ± SD (°)	*p* Value	Mean ± SD (°)	*p* Value	Mean ± SD (°)	*p* Value
Preoperative	13.09 ± 7.19		9.77 ± 5.37		14.52 ± 3.19	
PO	4.79 ± 4.58	<0.001 ^a^	4.13 ± 3.13	<0.001 ^a^	17.45 ± 2.23	<0.001 ^a^
PO1M	5.26 ± 4.08	<0.001 ^a^	4.06 ± 3.12	<0.001 ^a^	16.87 ± 2.19	<0.001 ^a^
PO3M	6.03 ± 4.18	<0.001 ^a^	4.34 ± 3.23	<0.001 ^a^	16.65 ± 2.31	<0.001 ^a^
PO6M	6.32 ± 4.27	<0.001 ^a^	5.10 ± 3.97	<0.001 ^a^	16.64 ± 2.56	<0.001 ^a^
PO1Y	6.83 ± 4.80	<0.001 ^a^	4.86 ± 4.25	<0.001 ^a^	16.68 ± 2.50	<0.001 ^a^
PO5Y	6.69 ± 4.46	<0.001 ^a^; 0.002 ^b^	4.88 ± 4.31	<0.001 ^a^; 0.283 ^b^	16.89 ± 2.50	<0.001 ^a^; 0.084 ^b^

Note: ^a^ Wilcoxon signed-rank test between preoperative and postoperative time points. ^b^ Wilcoxon signed-rank test between postoperative and PO5Y. SD, standard deviation.

**Table 3:** Implant position and migration distance on radiograph.

Timepoint	Ankle AP ^a^				Foot AP ^a^			
	Position ^b^	Mean ^c^ Migration (mm)	*IQR* (mm)	*p* Value	Position ^b^	Mean ^c^ Migration (mm)	*IQR* (mm)	*p* Value
Implant position								
PO ^d^	−4.46				+1.17			
PO1M	−1.55				+2.89			
PO3M	−1.77				+3.21			
PO6M	−1.73				+3.40			
PO1Y	−1.74				+3.53			
PO5Y	−2.05				+3.67			
Migration distance				**<0.001** ^e^				**<0.001** ^e^
PO–PO1M		3.24	1.68–4.6			3.01	1.25–3.63	
PO1M–PO3M		0.93	0.38–1.05			1.21	0.38–1.23	
PO3M–PO6M		0.49	0.1–0.7			0.54	0.1–0.73	
PO6M–PO1Y		0.51	0.1–0.73			0.64	0.1–0.9	
PO1Y–PO5Y		0.78	0.2–1.03			0.76	0.2–0.8	

Note: AP ^a^ = anteroposterior. Position ^b^ = implant tip extending medial to the talar axis was classified as negative, and extending laterally was classified as positive. Mean ^c^ = implant movement distance. PO ^d^ = postoperative. ^e^ = The Friedman test showed “PO–PO1M” has the greatest implant movement distance. Statistically significant *p* values are shown in bold.

**Table 4:** Summary of complications.

No.	Complication Type	Patient Details	Timing	Management	Outcome
1	Early implant dislodgement	13-year-old boy with low-energy ankle sprain	1 month	Revision STA with implant reinsertion	Complete symptom resolution was achieved with no sequelae, and the implant remained well-positioned at the 5-year follow-up
2	Implant subsidence	10-year-old female who received STA + Kidner procedure suffered from sprain injury	7 months	Lateral column lengthening	Bone union achieved at 1 year
3	Persistent sinus tarsi discomfort	8-year-old male without any trauma	12 months	Implant removal	Hindfoot and forefoot alignment were maintained with satisfactory correction at the 5-year follow-up
4	Persistent sinus tarsi discomfort	12-year-old male without any trauma	Symptoms persisted after surgery	Corticosteroid injection	Complete symptom resolution was achieved following corticosteroid injection at 6 months

## Data Availability

All data generated or analyzed during this study are included in this published article.

## Abbreviations

The following abbreviations are used in this manuscript:

STA	Subtalar arthroereisis
AP	Anteroposterior
SD	Standard deviation
IQR	Interquartile range
